# Human Papillomavirus Infection and Fertility Alteration: A Systematic Review

**DOI:** 10.1371/journal.pone.0126936

**Published:** 2015-05-18

**Authors:** Tiatou Souho, Mohamed Benlemlih, Bahia Bennani

**Affiliations:** 1 Laboratoire de Microbiologie et de Biologie moléculaire, Faculté de Médecine et de Pharmacie de Fès, Université Sidi Mohammed Ben Abdellah, Fez, Morocco; 2 Laboratoire de Biotechnologies, Faculté des Sciences Dhar El Mahraz, Université Sidi Mohammed Ben Abdellah, Fez, Morocco; 3 Equipe micro-organismes, génomique et Facteurs Oncogènes, Laboratoire de Pathologie Humaine, Biomédecine et Environnement, Faculté de Médecine et de Pharmacie de Fès, Université Sidi Mohammed Ben Abdellah, Fez, Morocco; University of Louisville School of Medicine, UNITED STATES

## Abstract

**Background:**

HPV is the most prevalent sexually transmitted infection and its effect in cancer induction is well documented. HPV infections are mostly asymptomatic, but it is unclear whether HPV infections can result in alterations of reproductive health.

**Objective:**

To determine the relationship between human papillomavirus infections and reproductive health in both men and women.

**Methods:**

A systematic literature review was performed in PubMed and ScienceDirect data bases from January 1994 through August 2014.

**Results:**

HPV infections are shown to be significantly associated to many adverse effects in the reproductive function. These adverse effects were reported in different levels from cells production to pregnancy and may be related to the infecting genotype.

**Conclusions:**

It appears from this study that HPV detection and genotyping could be of great value in infertility diagnosis at least in idiopathic infertility cases. Like for the risk of carcinogenesis, another classification of HPV regarding the risk of fertility alteration may be considered after deep investigations.

## Introduction

Human infertility is defined as the inability for a couple to conceive and produce offspring after at least twelve consecutive months of unprotected sexual intercourse. This definition seems insufficient because it does not take into consideration all the cases. In effect, for many couples, this inability can be overcome by a treatment or a medically assisted procreation procedure after diagnosis [1]. Infertility is a complex human health situation which particularly alters the quality of life in couples that face it. In 2010, around 1.9% and 10.5% of women in the reproductive age (20–44 years old) were affected by primary and secondary infertility respectively. High prevalence of infertility is noted in South Asia, Africa, Middle East, Central/Eastern Europe and Central Asia [2].

Infertility results from multiple factors that are responsible for impairments in the reproductive function in men and/or women. These factors include congenital and hormonal disorders, lifestyle, environmental hazards and psychological state. All these factors can lead to impairments in the function of genital organs, the production of reproductive cells, semen quality, sperm cells transport to oocyte, fertilization and embryo implantation steps [3–6]. In the case of many couples, the cause of infertility remains unknown.

Sexually transmitted infections (STI) can target different tissues along the genital tract in both men and women and lead to functional alterations. This can result in reduced fertility or even infertility. It is widely accepted that bacterial infections with *Neisseria gonorrheae*, *Treponema pallidum* and *Chlamydia trachomatis* can lead to fertility alterations [7–9]. The impact on reproduction alteration is suggested but not well understood in the case of some viral STI, such as human herpes virus (HSV), adeno-associated virus, human immune-deficiency virus (HIV), human cytomegalovirus (HCMV) and human papillomavirus (HPV) [10,11].

HPV is the most prevalent sexually transmitted infection and its genotypes can be divided into two different groups according to their ability to induce malignancy. Low-risk types are responsible for benign lesions such as genital warts and respiratory papillomatosis, whereas persistent infection with high-risk types can lead to malignant transformations in both the ano-genital and aero-digestive regions [12]. HPV infections are often asymptomatic and most of the time, people are infected without being aware. Even if the infection does not necessary lead to cellular lesions or proliferation, it is unclear whether HPV infections can silently lead to damages that alter the reproductive function. The answer to this question will determine the significance of HPV detection in infertility diagnosis and state if assisted reproduction procedures require specific managements for HPV positive patients.

In order to establish good practices on HPV management in reproduction health care, it is necessary to get an overview on relationships between HPV infection and fertility. The purpose of this work is to review the available knowledge regarding the implication of HPV infections in human reproductive health.

## Methods

A systematic literature search was performed in Pubmed and ScienceDirect data base platforms. The search was extended to the last two decades (from January 1994 through August 2014). The following search terms were used in combination with the term “human papillomavirus”: fertility, fertilization, infertility, miscarriage, sterility and reproductive health. Selected hits underwent abstracts screening in order to identify studies that were focused on HPV infection and reproductive health. Relevant articles were selected for full text reading. Articles that present results from studies on population based correlations between HPV and human reproductive health were systematically included. Retrospective as well as prospective studies in cross-sectional and controlled designs were taken into consideration. The papers that do not present comparison between HPV infected and HPV-negative groups were excluded.

The search also retrieved articles that are based on *in-vitro* experimentations to explain the effect of HPV infection on reproductive health. These articles were also included in order to get a deeper overview on mechanisms by which the virus could affect the reproductive health.

The data collected from selected articles were focused on three main topics: HPV prevalence in different groups; statistical associations between HPV prevalence and fertility outcomes in population based studies and main conclusions on *in vitro* experimentation studies.

This study was conducted according to the Preferred Reporting Items for Systematic Reviews and Meta-analyses (PRISMA) statement (S1 Checklist) [13].

## Results

**Fig 1:**
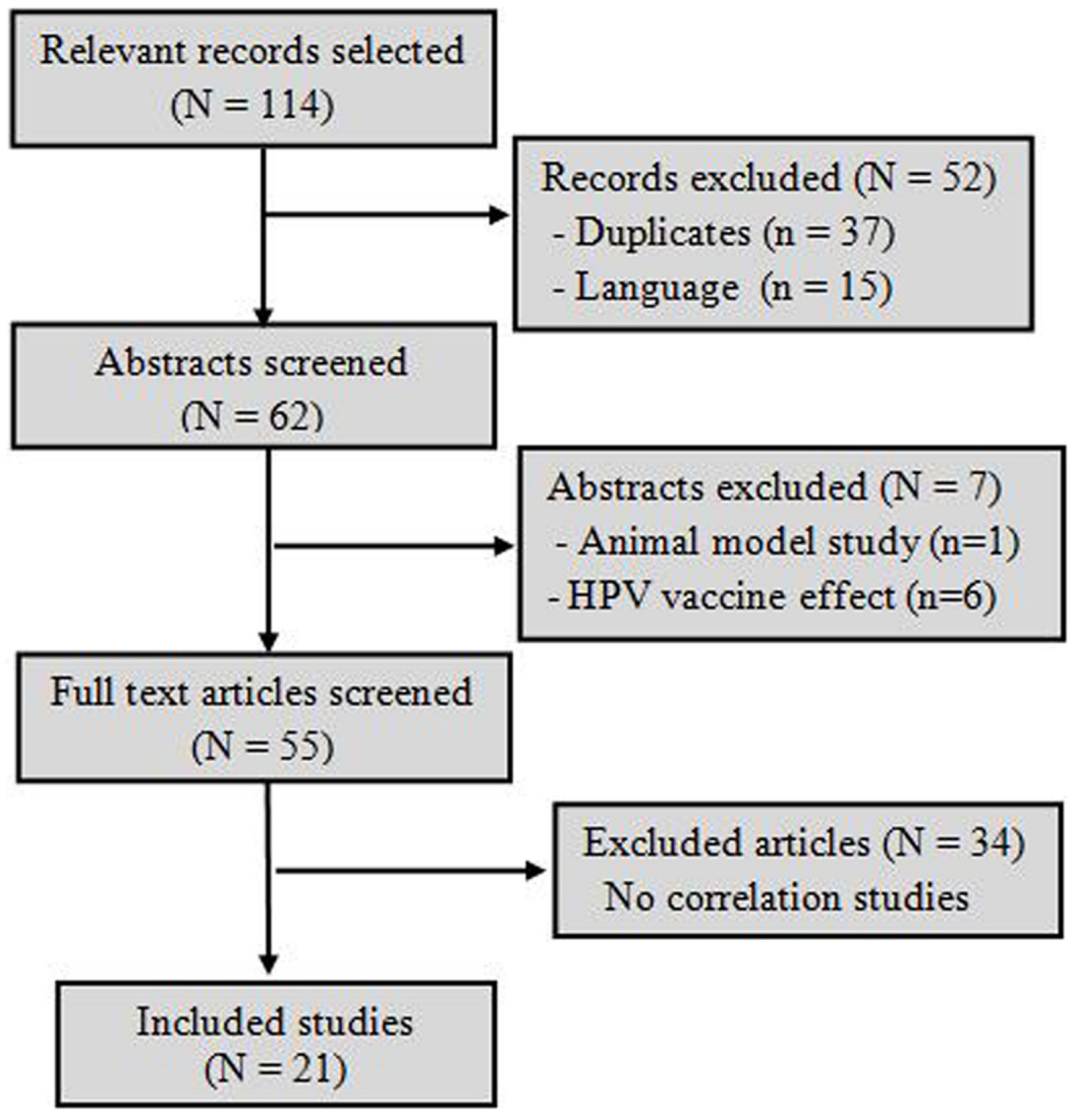
Studies selection data flow diagram.

The literature search retrieved a total of 1021 hits from which 114 records were selected for screening. Duplicated hits and articles that were not written in English or French were excluded. There remained 62 records that underwent the abstract screening. After abstract screening and full text reading, a total of 21 articles met our inclusion criteria. The whole search data flow diagram is presented in Fig 1.

It appears from this investigation that many research teams were interested in the possible interactions between HPV infection and the human reproductive health. Data from diverse studies about the relationship between HPV and reproductive health are now available and concern different aspects of the reproductive function. In effect, articles that met inclusion criteria were mainly focused on HPV infection and its relationship with semen parameters, *in vitro* fertilization or pregnancy outcomes. Authors mostly discuss the relationship between HPV and fertility parameters without giving special consideration to HPV persistence. Selected articles were pooled into three different groups according to the reproductive health aspect they are focused on:

- A group of nine (9) articles that studied the effect of HPV on semen parameters [14–22].
- A group of five (5) articles that are focused on the effect of HPV on *in vitro* fertilization outcomes [23–27].
- And a group of seven (7) articles that studied the effect of HPV infection on pregnancy outcomes [28–34].

## Discussion

### HPV infection and semen quality

It is now well established that HPV infections in men result in semen contamination. This contamination was suggested to be responsible for many alterations in semen quality [35]. In effect, *in-vitro* experimental studies show that exposure to HPV DNA induces DNA fragmentation in sperm cells. As in somatic cells, DNA fragmentation in spermatozoa is a signal that leads to apoptosis. It is noteworthy to consider that the effect of HPV DNA on semen could be genotype dependent. When exposure to DNA from HPV 16 or 31 leads to DNA fragmentation in sperm cells, nothing occurs when these cells are exposed to DNA from HPV types 18, 33 or 6 /11. In the same studies, it was also shown that DNA from HPV 16 or HPV 6/11 reduce more significantly the amplitude of sperm cells lateral head displacement compared to DNA from HPV types 18, 31 and 33 [14,15]. In addition to these observations, Lee et al. [14] recorded a significant decrease of sperm cells total motility in presence of DNA from HPV 6/11, 16, 18, 31 or 33 but Connelly et al. [15] found a contradictory result.

Several epidemiological studies revealed that HPV is more prevalent in infertile men or those with leukocytospermia compared to fertile ones [16, 18, 22]. It was shown in many cross-sectional and case-control studies that HPV infection is associated to poor semen quality. In effect, semen infection by HPV was significantly associated to reduced cell viability, reduced cell mobility, reduced amplitude of lateral head displacement, decreased cell count, decreased amount of normal morphology cells and the increased level of anti-sperm antibodies in semen (Table 1) [16–21].

**Table 1:** Epidemiological data of HPV effects on semen parameters.

Study	Study design	N	Sperm cells motility	Sperm cells count	Normal morphology cells	Anti-sperm antibodies
Yang Y et al., 2013 [16]	Case—control	1138	Decreased (p< 0.001)	Decreased ^#^	Decreased (p< 0.001)	
Garolla et al., 2013 [17]	Cross-sectional	257	Decreased (p< 0.01)	Decreased ^#^	Increased ^#^	Increased (p< 0.01)
Foresta et al., 2010 [18]	Cross-sectional	290	Decreased (p< 0.05)	Decreased (p< 0.05)	Decreased ^#^	
Foresta et al., 2010[19]	Cross-sectional	200	Decreased (p< 0.05)	Decreased ^#^	Decreased ^#^	
Lai et al., 1997 [20]	Cross-sectional	24	Decreased (P = 0.0416)			
Bezold et al., 2007 [21]	Case-control	241		Decreased (p< 0.05)		
Schilaci et al., 2013 [22]	Cross-sectional	308	Increased ^#^	Decreased ^#^	Increased ^#^	

^#^: The reported effect is a tend that did not reach the statistical significance

From the selected studies, an Italian cross-sectional study failed to find association between HPV and semen parameters [22]. This cohort of 308 men presents a particularly low HPV semen prevalence (7.8%) with predominance of HPV52. The absence of significant association can be explained by the reduced number of HPV-infected semen in comparison to HPV negative samples. However, it can also be hypothesized that the association between HPV and alterations of semen parameters may be specific for some genotypes as suggested by *in vitro* experiments.

### HPV and *in-vitro* fertilization

*In vitro* fertilization (IVF) and artificial insemination are medically assisted solutions for conception in many infertile couples. These approaches are proposed to couples for which semen parameters are altered or infertility is due to cervical hostility to spermatozoa. Considering the fact that HPV is associated with altered semen parameters, *in vitro* fertilization and embryo transfer could be considered a viable alternative in cases of reduced fertility associated with semen HPV infection.

It's well known that sperm cells adsorb HPV particles preferentially in two distinct sites along the equatorial region. This adsorption does not abrogate the fertilization potency of sperm cells but, it is unclear whether HPV infection has effect on steps following fecundation [36]. Therefore, studies on HPV association with IVF outcomes were included in our investigations.

Two distinct studies revealed a significant association between HPV infection and IVF failure. In the Italian study [23], the rate of IVF failure in HPV infected women was 40% vs. 13.5% in non infected women (p< 0.05). Similarly, it was found in USA a higher rate of IVF failure in HPV positive women (76.5% vs. 57%; p< 0.02) [24]. These studies provide epidemiological evidence that HPV is related to IVF failure. Nothing is known about the HPV genotype-specific action on IVF outcomes. This point was explored in Japan where the association between HPV 16 and IVF outcomes was studied [25]. This study concluded that HPV 16 is not associated to IVF failure. Others studies are needed to explore the influence of other HPV types and explain the mechanisms underlining HPV-related IVF failure.

Considering that HPV does not interfere in oocyte fertilization, the adverse effects of HPV in IVF may be explained by: i) cervical HPV-induced cytological lesions or ii) eventual alterations of the fetal health. The first hypothesis was explored in two different studies. Netherland study shows a significant association (p< 0.02) between HPV-induced cervical lesions and fertility reduction, the rate of high grade cervical lesions is two times higher in sub-fertile women eligible for IVF in comparison to the general population [26]. However, a study of 1044 Chinese women failed to find association between IVF outcomes and cytological abnormalities [27]. Therefore, more investigations are necessary to verify this hypothesis. The second hypothesis is discussed in the following section.

### HPV in female infertility and miscarriages

In comparison to studies related to HPV oncogenic effect in the female genital tract, there is a lack of studies on HPV effects in female fertility parameters such as hormonal balance or oocyte production. The most explored subject is the relationship that could exist between HPV infection and pregnancy outcomes. Most studies explored the subject epidemiologically but in vitro experimentations have been made to understand the effect of the virus in the fetal health. To highlight the effects of HPV on the pregnancy outcomes, we compared the results of studies that investigated the linkage between HPV infection and miscarriages or premature rupture of the membrane during pregnancy.

*In vitro* study of Gomez et al. [28] proved that trophoblasts transfected with plasmids harboring HPV16 genome undergo apoptosis at rates three to six times higher than trophoblasts transfected with empty plasmids. This apoptosis could be responsible for dysfunctions in placenta, reduction of embryo ability to invade the uterine wall and finally lead to miscarriages in the earlier stages of pregnancy or to membrane preterm rupture.

Case-control epidemiological studies were performed comparing the HPV prevalence of women who miscarried and that of controls. The results of the included studies are reported in Table 2. The association between HPV and miscarriages or premature rupture of membrane was proved in two distinct studies performed in USA. They compared HPV prevalence in placentas from women after term birth with spontaneous abortions, or with cases of preterm rupture of membrane [28, 29]. The association between HPV and miscarriages was not statistically confirmed in a study performed in Poland [30]. More investigations are required to better describe the effects of HPV during pregnancy and determine whether the association between HPV and miscarriages is genotype dependent. High-risk genotypes HPV 16 and 18 were not associated to miscarriages but nothing is known about the other genotypes [30].

**Table 2:** Relationship between HPV and miscarriages.

Study	Region	HPV infection site	Control		Spontaneous abortion cases		P value
			N	HPV prevalence (%)	N	HPV prevalence (%)	
Gomez et al., 2008 [28]	USA	Placentas	30	20	30	50	0.03
Hermomat et al., 1997 [29]	USA		15	20	25	60	0.0091
Skoczynski et al., 2011 [30]	Poland		78	24.4	51	17.7	0.366
Conde feraez et al., 2013 [32]	Mexico	Cervix	138	15.22	143	24.46	0.0538
Bennani et al., 2012 [33] ^a^	Morocco		713	41.1	38	68.4	0.001
Ticconi et al., 2013 [34] ^b^	Italy		475	61.89	49	26.53	< 0.001

^a^: HPV prevalence determined in women with history of spontaneous abortion (cases) or women without abortion history (control);

^b^: HPV prevalence determined in women with recurrent miscarriages (cases) or women without miscarriage history but with at least one term pregnancy (control).

When cervical HPV infection is considered instead of placental or abortive tissue infection, significant association is found between HPV positivity and premature rupture of membrane [31]. If HPV infection weakens the membrane, it could lead to miscarriages. In a case-control study performed in Mexico, HPV prevalence was higher in women experiencing spontaneous abortion than HPV prevalence in the control group but the difference did not reach the statistical significance [32]. However, it was shown that histories of abortion are significantly associated with high HPV prevalence [32, 33].

An Italian case-control study explored the possible role of HPV in recurrent miscarriages, comparing HPV prevalence in women with history of multiple miscarriages and in women with at least one pregnancy at term and no history of spontaneous miscarriages. The results show that HPV prevalence was higher in the group of women without a history of recurrent miscarriages. To explain this observation the authors hypothesized that: i) the immune reaction is potentially responsible for recurrent miscarriages and could be protective against cervical HPV infection ii) women with recurrent miscarriages were effectively HPV positive during their difficult pregnancies but the infection cleared more rapidly than women without recurrent miscarriages [34].

Thus, HPV infections can lead to adverse effects such as premature rupture of membrane or miscarriages. In fact, HPV trophoblasts infection corrupts the embryo’s health and its ability to invade the uterine wall. Even if cervical HPV infections can remain silent without cellular lesions, infected women can bear a healthy embryo but they should be paid more attention because of virus transmission to babies, a membrane premature rupture or miscarriage risks.

## Conclusion

HPV infection is well known for its ability to induce malignancy in the ano-genital and aero-digestive regions. The other aspect of HPV infection that deserves more attention is its association to damages that lead to reduced fertility or sterility. Both epidemiological and *in vitro* studies have been made to explore this subject.

It appears from this study that HPV can be associated to i) apoptosis in sperm cells; ii) alterations of semen quality through cell count decrease, amplitude of lateral head displacement reduction, mobility reduction and increase of anti-sperm antibodies level; iii) apoptosis in embryonic cells; iv) miscarriages or premature rupture of membrane.

If HPV is not the main cause, it must be considered as a risk factor of reduced fertility or infertility. Effectively, HPV detection in pregnant women or on their partners can be considered as a risk of preterm birth, miscarriages and virus transmission to the newborn. Thus, it will be useful to consider HPV detection in both men and women for infertility diagnosis and before IVF procedures.

Overall, some questions remain unanswered and many studies are required to clarify the mechanisms underlining the effects of HPV in both female and male reproductive system and to determine the role of HPV persistence in fertility alterations. In effect, we don't know if reduced fertility or infertility associated to HPV reflects transient effects and if total fertility will be restored after HPV clearance. Finally, as for cytological lesions induction, HPV genotype classification according to the risk of sterility and fertility reduction may be envisaged.

## Supporting Information

S1 Checklist PRISMA checklist. (DOCX)
